# Determining the active role of microscopists in community awareness-raising activities for malaria prevention: a cross-sectional study in Palawan, the Philippines

**DOI:** 10.1186/1475-2875-12-384

**Published:** 2013-11-01

**Authors:** Emilie LA Matsumoto-Takahashi, Pilarita Tongol-Rivera, Elena A Villacorte, Ray U Angluben, Junko Yasuoka, Shigeyuki Kano, Masamine Jimba

**Affiliations:** 1 Department of Community and Global Health, Graduate School of Medicine, The University of Tokyo, Tokyo, Japan; 2 Department of Parasitology, College of Public Health, University of the Philippines Manila, Manila, Philippines; 3 Kilusan Ligtas Malaria/Pilipinas Shell Foundation, Inc, Palawan, Philippines; 4 Department of Tropical Medicine and Malaria, Research Institute, National Center for Global Health and Medicine, Tokyo, Japan

**Keywords:** Malaria, Prevention, Microscopist, Community health workers, The Philippines, Structural equation modeling

## Abstract

**Background:**

Malaria remains one of the most prevalent and fatal diseases among the inhabitants of Palawan in the Philippines. Palawan, where healthcare services remain limited, has the highest malaria endemicity in the country. To eliminate malaria, effective prevention measures should be conducted alongside early diagnosis and prompt treatment, which are the major tasks of the trained microscopists in Palawan. However, while the microscopists have implemented community awareness-raising activities aimed at preventing transmission of malaria, the nature and quality of these activities have not been evaluated. The present study identified the factors associated with the strengthening of community awareness-raising activities for malaria prevention implemented by microscopists in Palawan.

**Methods:**

A cross-sectional study was conducted among 127 microscopists in Palawan. Data were collected using self-administered questionnaires from November 2010 to February 2011. For data analysis, structural equation modelling was conducted, based on the questionnaire results, to identify the impact of factors associated with the number of community malaria awareness-raising activities implemented by microscopists using the following assessment indicators: (1) place of assignment; (2) annual parasite index; (3) microscopists’ capacity (service quality, knowledge on malaria, and ability in malaria microscopy); (4) self-preventive measures against malaria; and (5) job satisfaction.

**Results:**

High microscopists’ capacity was found to be a significant factor for a greater number of community awareness-raising activities for malaria prevention. High microscopists’ capacity was significantly explained by its two sub-components: high service quality (active detection, diagnosis and treatment, prescription of anti-malarial, and follow-up) and high ability in malaria microscopy (preparation and documentation, slide preparation and observation, safe handling and disposal, and knowledge on the morphology of infected red blood cells).

**Conclusions:**

Microscopists’ capacity was identified as a significant factor in community awareness-raising activities for malaria prevention. Thus, the strengthening of service quality and ability in malaria microscopy should be of the highest priority.

## Background

With the emergence of drug-resistant parasites and vectors, malaria remains one of the world’s most important health issues [1]. As of 2010, there were approximately 3.3 billion people at risk of malaria living in 99 countries, with approximately 219 million cases of malaria and 660,000 deaths. The World Health Organization (WHO) estimated that approximately 80% of the cases occurred in regions of Africa and Southeast Asia, where healthcare services are limited.

In the Philippines, malaria is most endemic in Palawan, where it has (up until the time of writing) consistently ranked as one of the top 5 causes of morbidity [2-6]. Although the annual parasite index (API) per 1,000 decreased from 27.6 in 2004 to 13.0 in 2010. At the time of writing this manuscript, the annual number of cases in the island still exceeds 1,000.

In 1999, 344 community health workers in Palawan (one for each endemic village, excluding 76 non-endemic villages) were trained as malaria microscopists [2]. Using community health workers is a potentially inexpensive, effective and sustainable approach for bringing malaria treatment closer to homes [7,8]. It has particular application in rural areas, such as Palawan, where there is a recognized paucity of formal public and private healthcare providers.

Microscopists identify malaria infection and species of parasites, by microscopic examination of Giemsa-stained blood smears. Under the supervision of midwives, microscopists have administered first-line anti-malarial drugs to malaria patients. This community-based malaria control programme named *Kilusan Ligtas Malaria* (KLM), a Tagalog name that translates to “Movement Against Malaria,” has been maintained with the aid of the Japan International Cooperation Agency and the ongoing Global Fund Project through Pilipinas Shell Foundation, Inc. Activities run by KLM have included the basic malaria microscopy and refresher courses for microscopists, the hosting of an annual malaria conference and the maintaining of logistic measures.

To further reduce the endemicity of malaria in Palawan, in addition to providing early diagnosis and prompt treatment, microscopists are expected to conduct community awareness-raising activities for malaria prevention. In Palawan, there has been an ongoing yearly decrease in malaria morbidity and mortality since 1999, although the decrease in the rate of morbidity has slowed since 2006 [2-6]. It is now important for community members to take preventive measures on an individual basis and for microscopists to assist this by raising malaria awareness in their respective communities.

The primary objective of the present study was to identify the factors associated with the microscopists’ implementation of community awareness-raising activities with regard to both the types of activities and the frequency at which they were implemented. Since malaria incidence is very different in the northern and southern regions of the island, the second objective was to statistically clarify the regional differences of these factors. The findings would be useful for determining a strategy to further reduce the incidence of malaria in Palawan.

## Methods

### Study design and site

A cross-sectional study was conducted among the microscopists in Palawan, which is the fifth largest island in the Philippines. The island of Palawan is largely covered with tropical rainforest, and consists of 367 villages in 23 municipalities [9,10]. Its capital, Puerto Princesa City, is located at the center of the island and divides the island into the northern and southern regions. According to the Census of Population and Housing in 2010, the estimated total registered population was 1,025,800 (527,200 male and 498,600 female) [9,10]. The population is comprised of various ethnicities from the different indigenous groups in Palawan, including Cuyunon, Hiligaynon, Palawan, Cebuano, Ilocano, Bisaya, Kagayanan, and Tagbanwa. Some members of these indigenous groups do not speak Tagalog.

### Participants

Inclusion criteria were that, at the time of the survey, the participant was living in Palawan and that they were both registered and working as a microscopist. In this present study, the term “microscopist” refers to a community health worker who is trained as a microscopist and diagnoses malaria in febrile patients using a microscope, and prescribes first-line anti-malarial drugs when patients have malaria. Microscopists also implement community awareness-raising activities aimed at preventing transmission of malaria among their patients and their patients’ families. Before starting work, they were trained by trainers from a “Training of Trainers” programme conducted with malaria specialists from Japan and the Philippines [2]. In 2011, there were 290 registered microscopists, all of whom understood Tagalog.

### Data collection

The authors’ originally planned to recruit all 290 active microscopists in Palawan by contacting them at a malaria congress in the southern municipality (Brooke’s Point) in November 2010, and at the refresher courses held in the northern municipalities (Taytay and San Vicente) in February 2011. However, only 127 out of 290 active microscopists attended these seminars. The first and second data collection sessions recruited 81 participants and 46 participants, respectively. All attendees agreed to participate in the present study and provided written consent. The remaining microscopists could not attend the congress or the refresher course seminars due to transportation problems mainly because they were living in the remote islands or in the mountains such as Balabac, Busuanga, Coron, Culion, Linapacan, Quezon, and Rizal municipalities.

Self-administered questionnaires were handed out to all of the 127 attending microscopists. The literacy level among microscopists’ was considered sufficient to properly understand and answer all of the questions in the questionnaire because most of the microscopists (96.1%) had high school (48%), college (45.7%), or higher (3.1%) (Table 1). Of those who did not graduate from high school or college, three participants had not completed any grade of education (2.4%) and one (0.8%) had completed elementary school. The authors closely supervised all processes of data collection. However, all of the participants were able to read and answer the questionnaires by themselves and there were no inconsistencies in their responses.

**Table 1:** Socio-demographic status of participants with respect to place of assignment

**Socio-demographic status**	**Total (N = 127)**	**Northern region (n = 67)**	**Southern region (n = 60)**	**Chi-square test (*p*-value)**
**Age**	(Mean = 39.4, SD = 7.4, *p* = 0.691^a^)			
Low (≤39.4)	62 (48.8%)	35 (52.2%)	27 (45%)	0.368
High (>39.4)	64 (50.4%)	31 (46.3%)	33 (55%)	
**Gender**				
Man	13 (10.2%)	7 (10.4%)	6 (10%)	0.934
Woman	114 (89.8%)	60 (89.6%)	54 (90%)	
**Marital status**				
Never married	12 (9.4%)	6 (9%)	6 (10%)	0.518
Married	105 (82.7%)	56 (83.6%)	49 (81.7%)	
Divorced	2 (1.6%)	0 (0%)	2 (3.3%)	
Widowed	6 (93%)	3 (4.5%)	3 (5%)	
**Education**				
No grade completed	3 (2.4%)	2 (3%)	1 (1.7%)	0.404
Elementary	1 (0.8%)	0 (0%)	1 (1.7%)	
High school	61 (48%)	36 (53.7%)	25 (41.7%)	
College	58 (45.7%)	28 (41.8%)	30 (50%)	
Higher	4 (3.1)	1 (1.5%)	3 (5%)	
**Occupation**				
Homemakers	93 (73.2%)	49 (73.1%)	44 (73.3%)	0.408
Farmer: coconut	6 (4.7%)	1 (1.5%)	5 (8.3%)	
Farmer: rice	8 (6.3%)	5 (7.5%)	3 (5%)	
Fishery	15 (11.8%)	8 (11.9%)	7 (11.7%)	
Tourism-related	1 (0.8%)	1 (1.5%)	0 (0%)	
Midwife	2 (1.6%)	1 (1.5%)	1 (1.7%)	
Other	2 (1.6%)	2 (3%)	0 (0%)	
**Ethnicity**				
Bicolana	7 (5.5%)	4 (6%)	3 (5%)	*p* < 0.001 ***
Bisaya	28 (22%)	11(16.4%)	17 (28.3%)	
Ceuano	7 (5.5%)	5 (7.5%)	2 (3.3%)	
Cuyunon	33 (26%)	27 (40.3%)	6 (10%)	
Ilocano	5 (3.9%)	0 (0%)	5 (8.3%)	
Kagayan	3 (2.4%)	1 (1.5%)	2 (3.3%)	
Mindanao	2 (1.6%)	0 (0%)	2 (3.3%)	
Palawan	4 (3.1%)	0 (0%)	4 (6.7%)	
Tagalog	14 (11%)	6 (9%)	8 (13.3%)	
Tagbanwa	5 (3.9%)	0 (0%)	5 (8.3%)	
Mixed	4 (3.1%)	2 (3%)	2 (3.3%)	
Other	4 (3.1%)	1 (1.5%)	3 (5%)	
**Religion**				
Catholic	86 (67.7%)	52 (77.6%)	34 (56.7%)	0.006**
Christian except Catholic	35 (27.6%)	15 (22.4%)	20 (33.3%)	
Muslim	6 (4.7%)	0 (0%)	6 (10%)	
**Household wealth**^**1**^	(Median = 3, SD = 1.62, *p* = 0.000^a***)^			
Low (≤3)	59 (46.5%)	19 (31.7%)	40 (59.7%)	0.002**
High (>3)	68 (53.5%)	41 (68.3%)	27 (40.3%)	
**Duration of work as microscopist (month)**	(Median = 104, SD =38.7, *p* = 0.002^b***)^			
Low (≤104)	71 (55.9%)	25 (41.7%)	46 (58.7%)	0.002**
High (>104)	56 (44.1%)	35 (58.3%)	21 (31.3%)	
**Distance from home to nearest health center (min)**	(Median = 15, SD = 26.3, *p* = 0.796^c^)			
Low (≤15)	73 (57.5%)	32 (53.4%)	41 (61.2%)	0.371
High (>15)	54 (42.5%)	28 (46.7%)	26 (38.8%)	
**Reason for becoming microscopist**				
Voluntary	119 (93.7%)	60 (89.6%)	59 (98.3%)	0.042
Nominee	8 (6.3%)	7 (10.4%)	1 (1.7%)	

^1^This scale scores from 1–8 points as follows, with 1 point each for the following: electricity, radio, television, refrigerator, bicycle, motorcycle, bike-car, and tin or cement wall.

*Significant place of assignment difference (0.01 < *p* < 0.05), **Significant place of assignment difference (0.001 < *p* < 0.01), ***Significant place of assignment difference (*p* < 0.001).

^a^Independent *t*-test, ^b^Welch test, and ^c^Mann–Whitney *U* test were conducted to clarify the place of assignment difference between the northern and the southern regions.

### Measurements

**Figure 1:**
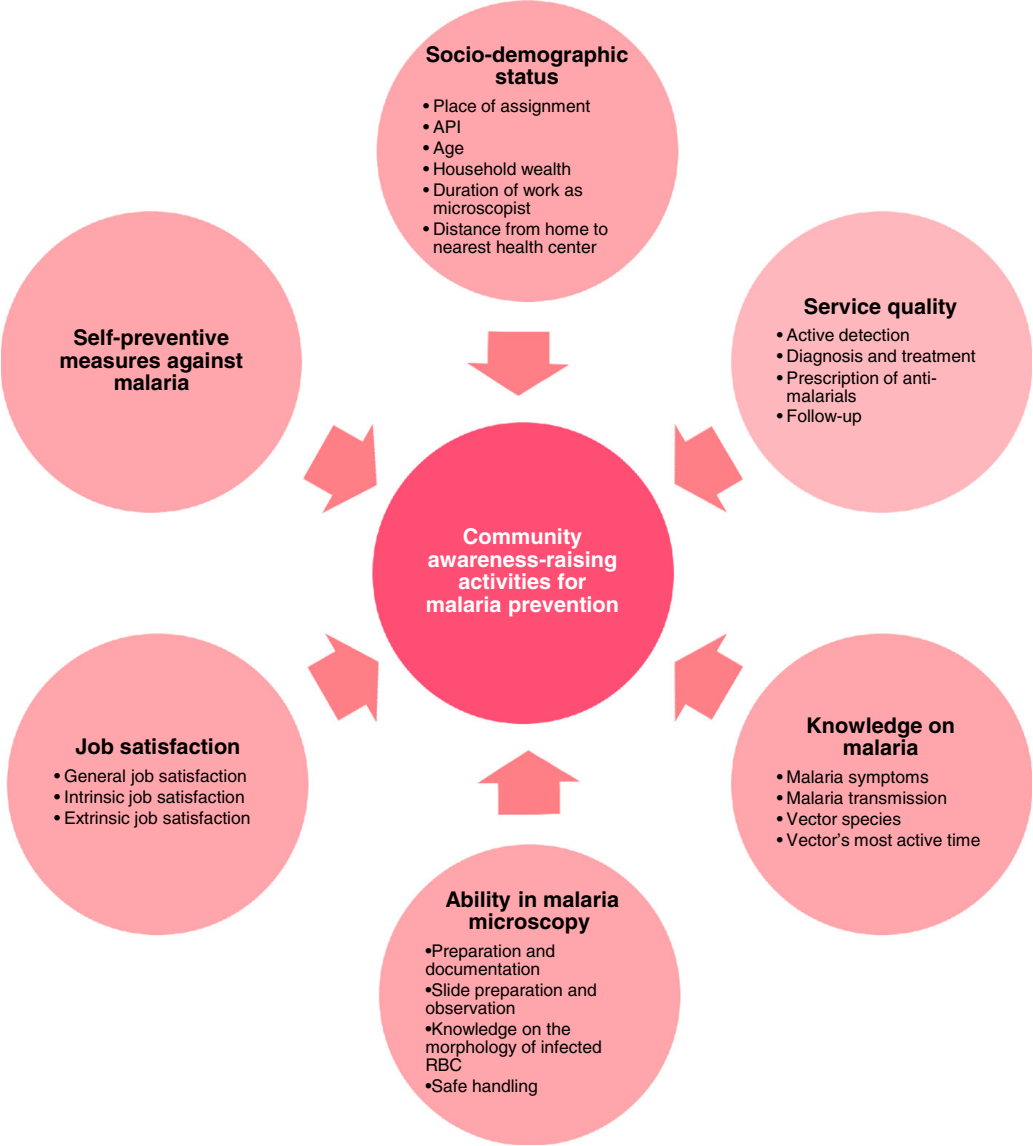
**Conceptual framework.** The authors hypothesize that an association exists between “community awareness-raising activities for malaria prevention” and the following multi-directional variables: “socio-demographic status”, “service quality”, “knowledge on malaria”, “ability in malaria microscopy”, “job satisfaction”, and “self-preventive measures against malaria”. Sub-variables of the each variable are in the circles of the conceptual framework. API, annual parasite index.

A structured questionnaire was developed (Figure 1). It included 134 questions regarding: (1) socio-demographic status; (2) community awareness-raising activities for malaria prevention; (3) service quality; (4) knowledge on malaria; (5) self-preventive measures against malaria; (6) ability in malaria microscopy; and (7) job satisfaction. Questions (2–5) were derived from the indices developed by Yasuoka *et al.*[11,12]. These indices were already used to measure the multi-dimensional quality of community malaria health workers in Cambodia. To measure (6) ability in malaria microscopy, a series of questions was developed based on the official training content for microscopists [13,14]. For (7) job satisfaction, the short form of the Minnesota Satisfaction Questionnaire was used. This questionnaire has been applied globally to measure the level of job satisfaction among health care professionals [15]. Additionally, regional data on demographic and malaria endemicity were also collected in the Provincial Health Office of Palawan. To enhance the validity of this questionnaire, it was reviewed by two local malaria experts who were knowledgeable on the situation of microscopists in Palawan.

### Socio-demographic status

The socio-demographic variables that were analysed included age, gender, marital status, educational status, ethnicity, religion, occupation, household wealth, duration of work as a microscopist, distance from their house to the nearest health centre, and reason for becoming a microscopist.

### Community awareness-raising activities for malaria prevention

Community awareness-raising activities are defined as activities to enhance a community’s knowledge on malaria and its prevention. The microscopists in Palawan perform community awareness-raising activities mainly among their patients and their patients’ families. They explain the process of malaria transmission and how to protect themselves from malaria. At such occasions, if necessary, the patients and their families are provided with WHO printed materials prepared by KLM staff [14].

To investigate the microscopists’ involvement in malaria prevention, the types and frequencies of community awareness-raising activities that they had implemented were measured. Microscopists were asked about the frequency of awareness-raising activities for eight preventive behaviors. Among the eight questions that were asked, six were about preventive behaviors for malaria infection, namely: “sleep inside bed nets,” “ bring mosquito nets to the forest,” “wear long-sleeve shorts/pants,” “fill out water pools,” “cover water jars/tanks,” and “spray house.” These questions included three response levels: “always (3),” “sometimes (2),” and “never (1).” The other two questions were about stigmatized attitudes: “should not come close to malaria patients” and “should not share utensils with malaria patients.” These questions also included three response levels, “always (1),” “sometimes (2),” and “never (3).” The total score of eight questions was treated as a continuous variable, and a higher score was interpreted as being indicative of greater activity in community awareness-raising activities.

### Service quality

Service quality was measured by asking questions on four assessment indicators: “active detection,” “diagnosis and treatment,” “prescription of anti-malarials,” and “follow-up.” In the case of “active detection,” the microscopists were asked about the regularity of home visits to detect malaria. Response levels ranged from “never (0)” to “regularly (3).” “Diagnosis and treatment” included five questions with three response levels ranging from: “never (0)” to “always (2).” For “prescription of anti-malarials,” four questions were asked, with response levels ranging from “never (0)” to “always (2).” “Follow-up” was measured by asking about the frequency of follow-up consultations with recovered patients and ranged from “never (0)” to “always (2).” Each of the four assessment indicators was divided by its maximum number of points, to give a maximum score of 1. The total score (range: 0–4) was treated as a continuous variable, and a higher total score was interpreted as being indicative of a higher quality of service from microscopists.

### Knowledge on malaria

To measure knowledge on malaria, four questions about malaria symptoms, six questions about malaria transmission, six questions about vector species, and four questions about most active time of vector were asked. The answer for each question was “correct (1),” or “incorrect (0).” As for the service quality index, each of the four assessment indicators was divided by its maximum number of points - giving a maximum of score of 1. The total score of these four assessment indicators (range: 0–4) was treated as a continuous variable.

### Self-preventive measures against malaria

Attitudes towards the performance of self-preventive measures against malaria were measured using five questions with three response levels ranging from “never (0)” to “always (2).” Responses indicated various preventive behaviors that the respondents had implemented, including: “come back home before dawn,” “wear long-sleeved shirts/pants,” “sleep inside bed nets at home,” refrain from going to the forest,” and “bring hammock nets to the forest”. A higher score was interpreted as indicating that a respondent had a greater tendency to perform self-preventive measures against malaria. The total score was treated as a continuous variable.

### Ability in malaria microscopy

Ability in malaria microscopy was measured with seven questions on “preparation and documentation,” 21 questions on “slide preparation and observation,” seven questions on “safe handling and disposal” of the smears, and 24 questions on “knowledge on the morphology of infected red blood cells (RBCs)” by *Plasmodium falciparum*, *Plasmodium vivax* and *Plasmodium malariae*. The sections “preparation and documentation,” “slide preparation and observation” and “safe handling and disposal” included three response levels: “always (2),” “sometimes (1),” and “never (0).” Regarding “knowledge on the morphology of infected RBCs,” eight questions were asked for each of the three species of parasite. The 24 questions could be answered with “correct (1)” or “incorrect “(0)”. The sum of the scores for all questions was calculated and the total score was treated as a continuous variable.

### Job satisfaction

To measure job satisfaction, the short form of the Minnesota Satisfaction Questionnaire was used. It included 20 questions with five response levels ranging from “very dissatisfied (1)” to “very satisfied (5).” A higher score indicated greater job satisfaction.

### Statistical analysis

After confirming the accuracy of the entered data with two authors, two types of statistical analysis were conducted. First, descriptive analysis was conducted to gain an overview of the characteristics of the participants. Second, structural equation modelling (SEM) was used to identify the factors associated with the number of community awareness-raising activities for malaria prevention. The correlation of all variables was examined and a path model was built based on the results of bivariate analysis (state model). The fit of the model was examined in terms of degree of freedom (df), chi-square (CMIN), comparative fit index (CFI), and root mean square error of approximation (RMSEA). According to conventional criteria, a good fit was indicated by CMIN/df < 2, CFI > 0.97, and RMSEA < 0.05, and an acceptable fit by CMIN/df < 3, CFI > 0.95, and RMSEA < 0.08 [16]. All statistical analyses were conducted using SPSS version 18.0 and Amos 18.0 (SPSS Inc., Chicago, IL, USA).

### Ethical considerations

All participants had a clear understanding of the principles of confidentiality and voluntary participation. Written consent was obtained from all participants before the questionnaires were distributed. The present study was approved by the Research Ethics Committee of the University of Tokyo and upheld by the Palawan Provincial Health Office.

## Results

### Population distribution, confirmed malaria cases, API and microscopist/region

Table 2 shows each region’s population, confirmed malaria cases and percentage of *P. falciparum* cases*,* annual parasite index (API) per 1,000 population, and the distribution of microscopists and participants per region in the year 2011. The majority (3,803 of 4,984, 76.3%) of malaria cases in Palawan were *P. falciparum.* The API of the southern region was approximately 20 times higher than that of the northern region (*p* < 0.0001). The API was especially high in the southern-most municipalities of Balabac (21.5), Quezon (19.4) and Rizal (33.2). In the majority of the northern municipalities, the API was < 1.

**Table 2:** Distribution of population, confirmed malaria cases, API, and microscopist/region

**Region**	**Population**	**Confirmed malaria cases (*P. falciparum *)**	**API**	**Microscopists**	**Participants**
**Total**	**872,390**	**4,984 (76.3%)**	**5.71**	**290**	**127 (43.8%)**
Northern Region	334,392	200 (57%)	0.60***	115	67 (58.3%)
Puerto Princesa City	207,119	795 (71.2%)	3.84	30	0 (0%)
Southern Region	330,879	3,989 (78.3%)	12.1***	145	60 (41.4%)

***Chi-square test between northern region and southern region (*p* < 0.0001).

In order to achieve an even distribution of microscopists, the organizers attempted to invite equal numbers of participants from the northern and southern regions. In total, 67 out of 115 northern microscopists (58.3%) and 60 out of 145 southern microscopists (41.4%) participated in the study.

### Socio-demographic status

Table 1 shows the results related to the socio-demographic status of respondents with respect to their places of assignment. To clarify the regional differences between northern region and southern region, in several socio-demographic variables, a Chi-square test was conducted. The participants’ ages ranged from 28–51 (mean 39.4 years, SD 7.4). The vast majority (about 90%) were female, of whom 82.7% were married. Forty-eight percent of the participants had graduated from high school and 48.8% had undertaken education beyond a high school level.

Homemakers comprised 73.2% of respondents. The remaining respondents were employed in jobs that included rice or coconut farmers, fishermen, in tourism-related businesses and midwives. The ethnicity and religion of respondents varied. Eleven ethnicities, with the Cuyunon and Bisaya indigenous groups making up the majority, are differently distributed from the northern to southern region (Chi-square test, *p* < 0.001). The majority of the participants were Christian (67.7% Catholic, 27.6% other denominations) and 4.7% were Muslim (*p* = 0.006). The Muslim respondents all came from the southern region of Palawan. Half of the participants had electricity, radio, television, and a house with tin or cement walls. About 20% had refrigerators, bicycles, and motorcycles. The participants from the southern region had higher household wealth than those from the northern region (*p* = 0.001).

The average duration of experience as a microscopist was 94.3 months (about 8 years). Most participants (76.4%) had become microscopists within three years of the start of the project. Microscopists in the northern region had greater experience than those in the southern region (*p* < 0.001).

The average distance from a microscopist’s home to the nearest health center (where most microscopists treat patients) was 21.2 minutes on foot (SD 38.7).

Most participants became microscopists voluntarily (93.7%). The reasons that they stated for becoming microscopists included: interest in reducing malaria in the village (66.1%), interest in saving villagers’ lives (6.3%), interest in malaria treatment and prevention (9.4%), and other reasons (11.9%). The remaining 6.3% of respondents had been nominated by community members or community leaders. The northern district had more nominated people than the southern region (*p* = 0.042).

### Community awareness-raising activities for malaria prevention

Differences in numbers were found in the community awareness-raising activities for malaria prevention that were reported to have been implemented by the microscopists. Almost all (99%) of the participants reported that they had undertaken at least one measure to prevent malaria infection in their community. More than 90% of the participants encouraged the community members to “sleep inside bet nets,” and “wear long-sleeve shirts/pants,” to avoid being bitten by infected mosquitoes and to “cover water jars and tanks.” Most participants (75.2%) reported always spraying their houses, while 22.4% reporting that they sometimes sprayed. However, some reported having passed on incorrect, stigma-based information: 15.7% told community members not to come close to malaria patients and 6.3% told community members not to share eating utensils with malaria patients. Only 68.5% of the participants explained the importance of bringing mosquito nets when people had to stay in the forest.

### Service quality

The average length of time per week that the participants spent for preventive activities was 18.2 hours (SD 24.9) in the dry season and 16.3 hours (SD 21.6) in the wet season. For curative activities, participants spent 9.4 hours per week (SD 18.0) in the dry season and 8.4 hours (SD 21.6) in the wet season. The geographical difference did not significantly influence the length of time spent on these activities.

The majority (93%) of participants were able to properly perform the basic and important task of making blood smears from febrile patients and diagnosing malaria infection. When they diagnosed people malaria positive, 87% of participants reported that they always gave anti-malarials.

As many as 91.3% of the participants reported that they either regularly or sometimes perform active detection. On a weekly basis, or sometimes several times per week, participants would visit patients in their community who had trouble with traveling to the nearest health center. Regarding prescription of anti-malarials, 91.3% of the participants successfully described the dosage and 92.9% of them could also explain the importance of compliance. A relatively small number (67.3%) explained to patients that compliance failure could result in incomplete treatment, while 88.2% could explain reasons for drug resistance. Regarding follow-up, 65% of the participants always checked if patients recovered and 89% reported that they always asked the patient’s family whether the patient had recovered satisfactorily.

### Knowledge on malaria

The percentages of participants who could obtain full scores for knowledge on malaria transmission, vector species and most active time of the vector were 57.9%, 63.5%, and 67.9%, respectively. It is notable that far fewer respondents were able to correctly respond to the questions on malaria symptoms, with only 35% obtaining a full score, and with 45.7% including diarrhea as a symptom of malaria. The malaria knowledge scores did not differ significantly between the northern and southern regions of Palawan.

### Self-preventive measures against malaria

The percentage of participants that reported always coming home before dawn was 63.8%, but the responses from the northern region (46.3%) and the southern region (83.3%) differed markedly (*p* < 0.01). The majority (92.1%) of respondents reported that they always wore long-sleeve shirts and pants to avoid mosquito bites; of these 88.1% were from the northern region, and 96.7% were from the southern region. Almost all of the respondents (97.6%) reported always sleeping inside bed nets at home, this included 95.5% of respondents from the northern region and all (100%) of respondents from the southern region. Approximately half (51.2%) of the respondents (35.8% from the northern region, 68.3% from the southern region) always refrained from going into the forest and if it was necessary to go into the forest, 69.3% (62.7% from the northern region, 76.7% from the southern region) reported that they always took a mosquito net. Participants from the southern region of Palawan were taking more preventive measures than participants from the northern region (*p* < 0.001).

### Ability in malaria microscopy

Table 3 shows the details and results of ability in malaria microscopy index (name of index, number of subscale, maximum score, content, participants’ mean score, SD, and accuracy rate). Most participants were able to perform the preparation and documentation, slide preparation and observation, safe handling and disposal of the blood smears, with each of the factors showing high mean values and satisfactory accuracy rates. The questionnaire for knowledge on the morphology of infected RBCs and the answers of participants are shown precisely in Table 4. Participants had a high ability to discriminate *P. falciparum.,* the most harmful species of the parasite, from the other parasite species from the characteristics of infected RBCs.

**Table 3:** Results of ability in malaria microscopy assessment indicators

**Assessment indicator**	**Microscopists (n = 127)**		
	**Mean**	**SD**	**Accuracy rate (%)**
**Ability in malaria microscopy (n = 4, maximum score = 5)**	**0.78**	**0.69**	**-**
**Preparation and documentation (n = 7, maximum score = 1)**	**0.9**	**0.11**	**-**
• Preparation of microscope, needle, methanol and first-aid dressings, Giemsa staining solution, slides and object slides	-	-	98
• Check the expiry dates of all solutions	-	-	74
• Write the names of the patient on the slides	-	-	92
• Write the date on each slide	-	-	63
• Select the 5th finger of the left hand to take the peripheral blood sample	-	-	92
• Clean the finger with alcohol swab and allow it to air dry	-	-	98
• Record the results in the CHW register	-	-	98
**Slide preparation and observation (n = 21, maximum score = 1)**	**0.76**	**0.09**	
• Take patient’s peripheral blood	-	-	98
• Prepare samples immediately after taking the blood	-	-	96
• Use clean slide	-	-	100
• Put one droplet of blood on the slide	-	-	79
• Using cover glass, spread the blood so as to obtain a thin layer of blood cells	-	-	97
• The angle of the cover glass is 30 degrees	-	-	72
• Dry immediately	-	-	99
• Fix with methanol for 2 to 5 minutes	-	-	94
• Too much drying damages the staining	-	-	26
• Keep the slides fixed with methanol horizontally and add the staining solution	-	-	82
• When numerous samples are used, use staining bottle	-	-	94
• Staining time depends on the concentration of the dyes(usually between 10 and 30 minutes)	-	-	88
• Maximum staining time is 45 minutes and even if you wait longer,the color does not change	-	-	49
• Wash with buffer	-	-	21
• If insoluble pigments are present at the surface of the solutions,take them off carefully	-	-	38
• Adjust the intensity of the staining through washing time with the buffer	-	-	17
• After washing, take off water quickly and dry with cold air	-	-	100
• Observe with microscope	-	-	100
• Nuclei of malaria parasite inside red blood cells will be stained in red	-	-	81
• The cytoplasm of malaria parasite inside red blood cells will be stained in blue	-	-	94
• When malaria parasite is found inside red blood cells, check the type of protozoa	-	-	97
**Safe handling and disposal (n =6, maximum score = 1)**	**0.92**	**0.15**	
• Put on a new pair of gloves when starting	-	-	67
• Do not touch patient blood	-	-	85
• Use a sterile lancet to puncture the patient finger	-	-	100
• Discard the needle in sharps bins immediately after usage	-	-	100
• Use a new needle for each patient	-	-	100
• Discard glove wrappers, alcohol swab, desiccant and cassette in non-sharps container	-	-	95
**Knowledge on the morphology of infected RBCs (n = 27, maximum score = 1)**			
Cited in Table 4	0.55	0.12	-

**Table 4:** Questionnaire on knowledge of the morphology of infected RBCs and answers of microscopists

**Point of discrimination**	***Plasmodium *** **species**					
	***P. falciparum***		***P. vivax***		***P. malariae***	
	**n**	**%**	**n**	**%**	**n**	**%**
**Size of infected red blood cells**						
Do not know	3	2.4	3	2.4	3	2.4
Small	49	38.6	5	3.9	44	34.6
Normal	**74**	**58.3**	4	3.1	**74**	**58.3**
Big	1	0.8	**114**	**89.8**	6	4.7
**Spots inside infected red blood cells**						
Do not know	5	3.9	4	3.1	14	11
Maurer’s dots	**104**	**81.9**	22	17.3	39	30.7
Schuffner’s dots	16	12.6	**98**	**77.2**	20	15.7
Ziemann’s dots	2	1.6	3	2.4	**53**	**41.7**
**Several parasites inside the same red blood cell**						
Do not know	5	3.9	10	7.9	11	8.7
Always	**72**	**56.7**	40	31.5	48	37.8
Sometimes	47	37	55	43.3	49	38.6
Never	3	2.4	**21**	**16.5**	**18**	**14.2**
**Parasite stages in the red blood sample**						
Do not know	8	6.3	7	5.5	20	15.7
Only the ring form	**59**	**46.5**	32	25.2	59	46.5
All stages	60	47.2	**87**	**68.5**	**44**	**34.6**
**Big ring structures**						
Do not know	8	6.3	7	5.5	9	7.1
Always	94	74	**88**	**69.3**	54	42.5
Never	**25**	**19.7**	32	25.2	**62**	**48.8**
**Chromatin dots**						
Do not know	9	7.1	9	7.1	13	10.2
Singular number	69	54.3	**82**	**64.6**	**82**	**64.6**
Plural number	**49**	**38.6**	35	27.6	30	23.6
**Band structures**						
Do not know	2	1.6	5	3.9	6	4.7
Always	48	37.8	34	26.8	**107**	**84.3**
Never	**77**	**60.6**	**88**	**69.3**	14	11
**Gametocytes in crescent form**						
Do not know	2	1.6	5	3.9	9	7.1
Always	**118**	**92.9**	9	7.1	19	15.0
Never	7	5.5	**113**	**89**	**99**	**78**

Correct answers are indicated with bold typeface.

### Job satisfaction

The average job satisfaction score in all participants was 83.4 (SD 8.9) out of 100 points. The participants reported that they were mostly satisfied to have the chance to contribute to their community: “the chance to do things for other people” (very satisfied/satisfied = 72.9%), “the chance to do something that makes use of my abilities” (very satisfied/satisfied = 67.3%), and “the chance to tell people what to do” (very satisfied/satisfied = 74.8%). The participants were also satisfied with the honor of being microscopists: “the chance to be “somebody” in the community” (very satisfied/satisfied = 64.8%), “the praise I get for doing a good job” (very satisfied/satisfied = 74.2%) and “the feeling of accomplishment I get from the job” (very satisfied/satisfied = 73%). They were satisfied with the way the jobs done: “the working conditions” (very satisfied/satisfied = 53.5%), “the chance for advancement in this job” (very satisfied/satisfied = 76.1%), “the way malaria control program policies are put into practice” (very satisfied/satisfied = 75.1%), and “the way my boss handles his/her workers” (very satisfied/satisfied = 61%). The lowest level of satisfaction noted among microscopists was in regard to the salary (high dissatisfaction 6.3%, dissatisfaction = 28.9%).

### Factors associated with community awareness-raising activities for malaria prevention

Bivariate analyses were conducted between all variables and several significant correlations were found (Table 5 and Table 6). A significant positive correlation was found between the number of community awareness-raising activities for malaria prevention and service quality, ability in malaria microscopy, and general job satisfaction (Table 5). The place of assignment (1 = northern region, 2 = southern region) was positively and significantly correlated with API (Pearson’s r = 0.76, *p* < 0.01), self-preventive measures against malaria (Pearson’s r = 0.31, *p* < 0.01), and general job satisfaction (Pearson’s r = 0.29, *p* < 0.01) (Table 6).

**Table 5:** Factors associated with community awareness-raising activities for malaria prevention

**Variables**	**r**	**Pearson’s correlation**
		***p-*** **value**
**Socio-demographic status**		
API	0.09	0.303
Age	−0.02	0.856
Household wealth	0.70	0.435
Duration of work as microscopist	−0.12	0.197
Distance from home to nearest health center	−0.19	0.035*
**Service quality**	**0.21**	0.035*
Active detection	0.17	0.064
Diagnosis and treatment	0.19	0.030*
Prescription of anti-malarials	0.01	0.883
Follow-up	0.11	0.204
**Self-preventive measures against malaria**	**0.16**	**0.067**
**Ability in malaria microscopy**	**0.27**	0.003**
Preparation and documentation	0.26	0.003**
Slide preparation and observation	0.17	0.055
Knowledge on the morphology of infected RBCs	0.16	0.073
Safe handling	0.09	0.343
**Knowledge on malaria**	**0.00**	**0.980**
Malaria symptoms	0.09	0.328
Malaria transmission	−0.19	0.034*
Vector species	0.04	0.679
Vector’s most active time	0.25	0.004**
**Job satisfaction**		
General job satisfaction	**0.92**	0.031*
Intrinsic job satisfaction	0.15	0.095
Extrinsic job satisfaction	0.20	0.027*

*Significant place of assignment difference (0.01 < *p* < 0.05), **Significant place of assignment difference (0.001 < *p* < 0.01), ***Significant place of assignment difference (*p* < 0.001). API, annual parasite index.

**Table 6:** Correlation matrix

		**1**	**2**	**3**	**4**	**5**	**6**	**7**	**8**
**1**	Community awareness-raising activities for malaria prevention	1							
**2**	Place of assignment (1 = northern region, 2 = southern region)	0.05	1						
**3**	Annual parasite index	0.09	0.76**	1					
**4**	Service quality	0.21*	−0.02	0.05	1				
**5**	Knowledge on malaria	0.00	−0.05	−0.13	0.17*	1			
**6**	Self-preventive measures against malaria	0.16	0.31**	0.19*	0.08	0.16	1		
**7**	Ability in malaria microscopy	0.27**	−0.70	−0.06	0.31***	0.09	0.05	1	
**8**	General job satisfaction	0.92*	0.29**	0.32**	0.21*	0.01	0.20*	0.24**	1
	Mean	2.59	-	7.11	17.70	8.38	4.19	72.53	84.00
	SD	0.19	-	8.19	2.60	1.72	0.89	6.28	8.77
	Skewness	1.67	-	1.15	−0.97	−1.05	−0.76	−0.50	−0.31
	Cronbach’s α	0.733	-	-	0.581	0.545	0.747	0.667	0.857

*Significant place of assignment difference (0.01 < *p* < 0.05), **Significant place of assignment difference (0.001 < *p* < 0.01), ***Significant place of assignment difference (*p* < 0.001).

**Figure 2:**
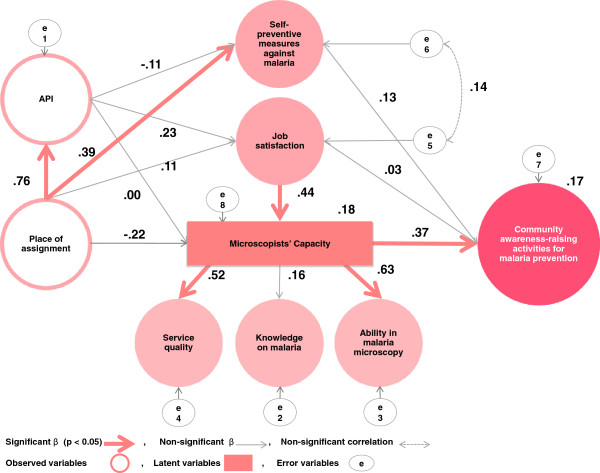
**The path diagram.** Chi-square = 12.667, df = 13, CFI = 1.000, RMSEA = 0.000. Place of assignment is calculated as follows: the northern region is “1” and the southern region is “2.” API, annual parasite index.

Based on these bivariate analyses, a hypothetical SEM was built to examine the relationship between community awareness-raising activities and other variables. The hypothetical SEM was selected from several models, with consideration of fitness between the data and the model, and of the usability obtained from the results. The latent variable was assembled from three similar observable variables. An upper latent variable (“microscopists’ capacity”) was set on “service quality,” “knowledge on malaria,” and “ability in malaria microscopy,” because these three indices account for the “microscopists’ capacity” (Figure 2). The use of the latent variable promotes greater efficiency and productivity in analysis than directly using multiple observable variables. The correlations between “service quality” and both “knowledge on malaria” and “ability in malaria microscopy” were found to be significantly high (*p* < 0.05 and *p* < 0.001, respectively) (Table 6).

The results of the SEM are illustrated in Figure 2. In this model, the following directional paths were drawn: from place of assignment to API, microscopists’ capacity, general job satisfaction, and self-preventive measures against malaria; from API to microscopists’ capacity, general job satisfaction, and self-preventive measures against malaria; from general job satisfaction to microscopists’ capacity; from microscopists’ capacity to ability in malaria microscopy, knowledge on malaria, and service quality; from microscopists’ capacity, general job satisfaction, and self-preventive measures against malaria to community awareness-raising activities. Bi-directional paths from general job satisfaction to self-preventive measures against malaria were drawn.

The hypothetical SEM fit the data: CMIN/df = 0.97, CFI = 1.000 and RMSEA = 0.000 (Figure 2). It revealed that the only significant and positive association between community awareness-raising activities was with the microscopists’ capacity (path coefficient = 0.37, *p* < 0.05). Microscopists’ capacity explained service quality (path coefficient = 0.52, *p* < 0.05) and ability in malaria microscopy (path coefficient = 0.63, *p* < 0.05). Job satisfaction explained microscopists’ capacity (path coefficient = 0.44, *p* < 0.05), but failed to explain community awareness-raising activities. The place of assignment only impacted API (path coefficient = 0.76, *p* < 0.05), which was twenty times higher in the southern region of Palawan, and the self-preventive measures against malaria (path coefficient = 0.39, *p* < 0.05), but did not impact the microscopists’ capacity or job satisfaction.

## Discussion

The results of the present study showed that the quality of service from microscopists in Palawan was high, but that some minor corrections of performance are necessary on an individual-by-individual basis. Regarding service quality, although participants were trying to spend more time to improve the health conditions of their communities, the rainy season and topography might have inhibited their activities due to associated transportation difficulties. Efforts to create better road conditions might be financially difficult, but the building of higher-quality infrastructure to facilitate reliable transportation could have a positive impact on the health of the inhabitants of Palawan. It should be noted, however, that the high follow-up coverage did not differ significantly between the northern and southern regions of the island. This might be indicative of close community relationships in Palawan - an intimacy which could play a substantial role in the microscopist intervention.

Inadequate knowledge on malaria remains a matter of concern. Only 68.5% of the participants explained the importance of bringing mosquito nets when people had to stay in the forest. This is a matter that should be addressed, given that most malaria patients from the southern region of the island were suspected to have been infected while working in the forest. In particular, microscopists are required to improve the self-preventive measures of people going to the mountains for taking care of their rice paddies or fields, or working in the mines.

The job satisfaction of the microscopists was high in all respects except for that of salary. The work is basically unpaid, however, several municipalities give differing financial incentives, mainly to cover the cost of transportation. Some microscopists were using their own money to travel for active case detection and treatment. A strategy for improving this situation is considered necessary.

In the present study, the results of SEM analysis indicated that community awareness-raising activities were solely and significantly influenced by high microscopists’ capacity (service quality and ability in malaria microscopy). General job satisfaction had a significant and positive influence on microscopists’ capacity. Consequently, this has remotely influenced the community awareness-raising activities for malaria prevention. Regarding the microscopists’ places of assignment, the present study found that it only impacted API and self-preventive measures against malaria. The findings indicated that high microscopists’ capacity was the sole factor associated with a greater number of community awareness-raising activities for malaria prevention. Enhancement of microscopists’ capacity is the key to strengthening the community awareness-raising activities for malaria prevention that they undertake. The results also suggest a noteworthy possibility: that, with regard to microscopists’ capacity, service quality and ability in malaria microscopy might be more important than knowledge on malaria. Since both service quality and ability in malaria microscopy among microscopists on Palawan were quite high, major interventions will not be needed to improve the microscopists’ effectiveness. These improvements may be achieved with only minor corrections that target the strengths and weaknesses of individual microscopists in Palawan. One possible area for that could be strengthened is diagnostic accuracy. While participants were able to differentiate *P. falciparum* from other forms of *Plasmodium*, but detailed identification of the other species could be better promoted.

The present study supports the impact of job satisfaction on work performance. Although no research has been done with microscopists, the effect of job satisfaction has been investigated in relation to performance/productivity, demission/career change, and absence [17-19]. The correlation between job satisfaction and productivity is reported to be greater for those in professional jobs [20]. The position of microscopist in Palawan is a professional job that requires special skills, thus this might have impacted the outcome. Moreover, another study reported that, especially in women, job satisfaction was negatively correlated with workplace absences not due to sickness [21]. Since the majority of the microscopists were women, the relationship between job satisfaction and performance may be strengthened. If a person is satisfied with his or her job, this satisfaction presumably leads to better job quality. In any type of health facility, the mission is to achieve the highest attainable level of medical practice. For this purpose, employee job satisfaction among health facility staff is very important, not only for employee wellbeing but also for the health facility and the community. The job satisfaction of microscopists and other community health workers should be the subject of greater attention and emphasis.

The place of assignment was only linked with API and self-preventive measures against malaria. It was assumed that people who lived in highly endemic areas were taking more self-preventive measures against malaria than those who lived in less endemic areas. The microscopists in the southern region had many more duties to perform than those in the northern region, but geographic location was found to have no significant impact on job satisfaction, microscopists’ capacity, and community awareness-raising activities for malaria prevention. This may be verified by the quality of the training program for microscopists. Alternatively, job satisfaction, microscopists’ capacity and involvement in community awareness-raising activities could be determined, not by the scale of the job, but by other elements such as trust or respect from the community. Further studies are needed to determine the effects of these elements.

In 2009, the API was 20 times higher in the southern region of the island than it was in the north. These results indicated that the high activity-level of the microscopists in the southern region was high. However, they also indicated that appropriate treatment and diagnosis alone is not sufficient to achieve a decrease of malaria in the southern region. The strengthening of malaria preventive measures in the southern region is still necessary and thus the implementation of community awareness-raising activities by microscopists should be of the highest priority.

The limitations of the present study should be noted. Firstly, the nature of a cross-sectional study design should be considered. Even though our model was highly suited to the data, the possibility of an opposite directional path cannot be ruled out. For example, the directional path from job satisfaction towards microscopists’ capacity might be opposite or dual-directional (correlation). Further longitudinal research might be needed to examine the causality. The method of data collection was the second limitation. It was not possible to conduct a random sampling because of the difficult geographical situation and security problems in Palawan. Microscopists who did not participate in the present study were mainly living in the remote islands of the province and had transportation problems. However, few malaria cases are reported from the remote islands, and it could be said that the present study was able to obtain the information from areas with a greater malaria burden. The third limitation was that the Cronbach’s alpha reliability coefficients of “service quality index” and “self-preventive measures against malaria index” were relatively low. The results implied that service quality and self-preventive measures against malaria varied by individual, but further research is needed to explore this issue.

## Conclusions

In the present study, the predictors of community awareness-raising activities for malaria prevention by microscopists in Palawan were identified. Microscopists’ capacity was found to be a significant factor for community awareness-raising activities. The significance of microscopists’ capacity can be explained by its two sub-components: service quality and ability in malaria microscopy. Job satisfaction also explained microscopists’ capacity; however, it did not affect community awareness-raising activities. Minor corrections depending on the strengths and weaknesses of individual microscopists are necessary in order to improve service quality and ability in malaria microscopy. The implementation of such corrections is an intervention that might succeed, not only in achieving an improvement in microscopists’ capacity, but also in achieving an increase in the number of community awareness-raising activities for malaria prevention that take place in the communities of Palawan. These findings point towards the possibility of implementing some relatively simple, low-cost interventions to boost the effort to reduce the number of malaria cases in Palawan.

## Abbreviations

API: Annual parasite index; CFI: Comparative fit index; CMIN: Chi-square; RMSEA: Root mean square error of approximation; KLM: *Kilusan Ligtas Malaria* (Tagalog), [Movement against Malaria]; RBCs: Red blood cells; SEM: Structural equation modelling; SD: Standard deviation; WHO: World Health Organization.

## Competing interests

The authors declare that they have no competing interests.

## Authors’ contributions

ELAM-T, JY, SK and MJ drew the study. ELAM-T, PTR, EAV and SK conducted the fieldwork and collected the data on the island. RUA helped to get the information on-site. ELAM-T, SK and MJ analysed the data, and ELAM-T wrote this paper under the supervision of SK and MJ. All authors read and approved the final manuscript.
